# Alterations in early cytokine-mediated immune responses to *Plasmodium falciparum *infection in Tanzanian children with mineral element deficiencies: a cross-sectional survey

**DOI:** 10.1186/1475-2875-9-130

**Published:** 2010-05-17

**Authors:** Erasto V Mbugi, Marjolein Meijerink, Jacobien Veenemans, Prescilla V Jeurink, Matthew McCall, Raimos M Olomi, John F Shao, Hans Verhoef, Huub FJ Savelkoul

**Affiliations:** 1 Cell Biology and Immunology Group, Wageningen University, The Netherlands; 2 Host-Microbe Interactomics, Wageningen University, The Netherlands; 3 Danone Research, Wageningen, The Netherlands; 4 Department of Medical Microbiology, Radboud University, Nijmegen, The Netherlands; 5 Kilimanjaro Christian Medical Centre (KCMC), Moshi, Tanzania; 6 London School of Hygiene and Tropical Medicine, Nutrition and Public Health Intervention Research Unit, London, UK; 7 Muhimbili University of Health and Allied Sciences, Biochemistry Department, School of Medicine, Dar es Salaam, Tanzania

## Abstract

**Background:**

Deficiencies in vitamins and mineral elements are important causes of morbidity in developing countries, possibly because they lead to defective immune responses to infection. The aim of the study was to assess the effects of mineral element deficiencies on early innate cytokine responses to *Plasmodium falciparum *malaria.

**Methods:**

Peripheral blood mononuclear cells from 304 Tanzanian children aged 6-72 months were stimulated with *P. falciparum*-parasitized erythrocytes obtained from *in vitro *cultures.

**Results:**

The results showed a significant increase by 74% in geometric mean of TNF production in malaria-infected individuals with zinc deficiency (11% to 240%; 95% CI). Iron deficiency anaemia was associated with increased TNF production in infected individuals and overall with increased IL-10 production, while magnesium deficiency induced increased production of IL-10 by 46% (13% to 144%) in uninfected donors. All donors showed a response towards IL-1β production, drawing special attention for its possible protective role in early innate immune responses to malaria.

**Conclusions:**

In view of these results, the findings show plasticity in cytokine profiles of mononuclear cells reacting to malaria infection under conditions of different micronutrient deficiencies. These findings lay the foundations for future inclusion of a combination of precisely selected set of micronutrients rather than single nutrients as part of malaria vaccine intervention programmes in endemic countries.

## Background

In African populations, multiple micronutrient deficiencies, infections and immunodeficiencies commonly co-exist. Deficiencies in vitamins and mineral elements can impair immune responses to infectious diseases through multiple mechanisms, ranging from phagocytosis and innate immune responses to antibody formation and cell-mediated immunity. Zinc is an important micronutrient because it is essential for the development, differentiation and function of several critical types of immune cells [1,2]. *In vitro *mitogen stimulation experiments indicate that marginal zinc deficiency can cause reduced counts of circulating leucocytes and reduced whole blood concentrations of cytokines, particularly IL-6 [3]. Zinc deficiency contributes to pneumonia, acute and chronic diarrhoea [4,5], and possibly malaria [4-6], which together constitute the leading causes of death in African children. In addition, zinc deficiency may exacerbate the outcome of diseases such as HIV and tuberculosis that rely on macrophage killing of infected cells [7]. Deficiencies of copper [8], iron and vitamin B_12 _have been associated with impaired neutrophil functions whereas deficiencies of folic acid are not [9].

A fast-acting innate immune response, mediated by cytokines such as interleukine-1β (IL-1β), IL-12 and tumour necrosis factor (TNF), is crucial for host survival in the initial stages of *Plasmodium falciparum *infection [10]. Zinc is needed for monocytes and macrophages to produce IL-1β and for other peripheral blood mononuclear cells (PBMCs) to produce TNF-α. Zinc deficiency can lead to impaired phagocytosis and intracellular killing by macrophages and neutrophils. In addition, it can impair NK-cell function, cytokine production, the generation of an oxidative burst as well as complement activity [2,11-14] through decreased activation of various cellular responses and low concentrations of IL-1β. In addition, innate immune responses determine the type and efficiency of subsequent adaptive immune responses [10,15,16] at later stages of infection.

This study was conducted to assess the impact of deficiencies of zinc and other mineral elements on early innate immune responses to *P. falciparum *infection. This micronutrient deficiency was assessed *in vitro *by stimulation experiments, using PBMCs samples that were collected from Tanzanian children aged 6-72 months. The assumptions were that zinc deficiency alters the balance in cytokine production and their association in early immune responses, and that deficiencies of zinc and other mineral elements induce a decreased ability of PBMCs to produce pro-inflammatory cytokines, and the regulatory cytokine IL-10, when exposed to *P. falciparum *parasites. In addition, it was analysed to what extent the magnitude of the PBMCs cytokine response depended on the *P. falciparum *infection status of the child at the time that the blood was collected and PBMCs were isolated.

## Methods

### Study area and population

This study was conducted in a lowland area around Segera village (S 05° 19.447', E 38° 33.249'), Handeni District, north-eastern Tanzania, in May-July 2006. Malaria is highly endemic in this area, with virtually all infections being due to *P. falciparum*. The residents in the study population mostly comprise poor farmer families growing maize and cassava for subsistence use. Such populations are prone to deficiencies of zinc and iron because they have cereal-based diets that are rich in natural dietary constituents that inhibit the absorption of these trace metals [17]. At the time of our study, only one health centre in Segera was available to serve all of the surrounding area. The study was approved by Ethics Review Committees in The Netherlands and Tanzania (reference numbers for KCMC and the National Health Research Ethics Review sub-Committee: 094 and NIMR/HQ/R.8a/VolIX/540, respectively). Informed consent was obtained from community leaders and local government officials, and from parents or guardians.

### Sampling methods and eligibility criteria

A census list was made with all resident children aged 6-72 months in the study area. Using this list, 16 children were randomly selected from 19 communities, resulting in a total of 304 subjects. Further details are provided elsewhere [18].

### Field procedures

All children were examined by a clinical officer, who also measured axillary temperature by electronic thermometer. Subjects were eligible when they had no fever, and showed no signs of other severe disease or severe malnutrition (weight-for-height z-score below -3 SD). *Plasmodium *infection was detected both by microscopy and rapid immunochromatographic assay (Vista Diagnostics Int. Kirkland, WA, USA, based on antibodies developed for OptiMAL test by Flow Inc., Portland, OR, USA). Although this dipstick test cannot be used to determine the duration of infection, it detects lactate dehydrogenase (pLDH) produced by live parasites only, either *P falciparum *or any human *Plasmodium *species [19,20]. For blood samples with > 50 *P. falciparum *parasites/mL (0.001% parasitaemia), it has been found that the OptiMAL assay has a sensitivity of approximately 96% [20]. Venous blood (6 mL) was collected in containers suitable for mineral element analysis with sodium heparin as anticoagulant (Becton-Dickinson, Franklin Lakes, NJ). Immediately upon collection, the cap was sprayed with ethanol and allowed to dry; approximately 1.3 mL blood was then drawn by sterile syringe. This aliquot was centrifuged and plasma samples were stored and transported to The Netherlands at -80°C for subsequent measurement of mineral element concentrations. The remainder of the blood sample was kept at 20-25°C during transport the same day to the laboratory in Moshi, at approximately 300 km distance, for collection of additional plasma and PBMCs. Children were treated for common childhood infections and anaemia according to guidelines of Tanzanian Ministry of Health.

### Determination of plasma concentrations of mineral elements

Plasma samples were diluted 20 times in milliQ [21], and concentrations of zinc, magnesium and copper were measured by inductively-coupled plasma atomic emission spectrometry (ICP-AES) (Vista Axial, Varian, Australia). To determine variability in outcomes, measurements were replicated five times. With mean values set at 100%, measurements varied between 97% to 102% for zinc, 99% to 102% for magnesium, and 97% and 102% for copper. Because we found no evidence for copper deficiency as assessed by plasma copper concentrations < 7.1 μmol/L (unpublished data), only the results for zinc and magnesium are reported in this paper.

### Determination of plasma indicators of iron stores and inflammation

After arrival at the laboratory in Moshi, blood samples were immediately centrifuged (300 × *g*) at ambient temperatures for 10 minutes. Plasma (1.2 mL) was collected and replaced this immediately with an equal volume of Iscove's modified Dulbecco's medium (IMDM) with GlutaMAX (Invitrogen Gibco-BRL, Life Technologies, Grand Island, NY, USA) for subsequent isolation of PBMCs (see below). Plasma was stored in liquid nitrogen, and subsequently transported on dry ice to The Netherlands, where plasma concentrations of ferritin and C-reactive protein were measured as indicators of iron stores and inflammation, respectively, by using a Behring nephelometer (BN ProSpec; Dade-Behring) in The Netherlands (by dr. J. P. M. Wielders at the Meander Medical Centre, Amersfoort, the Netherlands).

### PBMCs isolation

PBMCs were isolated by Ficoll density gradient centrifugation, cells were transferred to 10% v/v DMSO in fetal calf serum, cooled at -1°C/minute in an isopropyl-loaded device (Nalgene, Rochester, NY, USA) and preserved in liquid nitrogen [22]. For a 16-h period during transport to Wageningen University, The Netherlands, the PBMCs were kept on dry ice, and immediately thereafter stored again in liquid nitrogen until stimulation experiments.

### Preparation of *P. falciparum*-parasitized and unparasitized erythrocytes

Routinely prepared asexual stage parasitized erythrocytes were obtained from the Department of Medical Microbiology, Radboud University, Nijmegen [23]. Briefly, human O and rhesus-negative erythrocytes from healthy blood donors (Sanquin, Nijmegen, The Netherlands) were cultured in medium to which live *P. falciparum *parasites (NF54 strain) produced in a continuous culture were added [23,24]. After two to four days, when ~8-10% of erythrocytes were parasitized by asexual *Plasmodium *stages, the culture was concentrated by centrifugation at 625 × *g *for 5 min; parasitized erythrocytes were separated on a 67% Percoll gradient as reported elsewhere [25] and washed twice in phosphate-buffered saline (PBS). Purified parasitized erythrocytes were preserved at a concentration of approximately 15 × 10^7^/mL in 13% glycerol/PBS in a freezing container at -80°C. Glycerol (50% w/v) was added to the parasitized erythrocytes to avoid mechanical damage of the cells through ice formation. Unparasitized erythrocytes were processed similarly but without adding parasites to serve as a control. Both in parasitized and unparasitized erythrocyte cultures, we confirmed the absence of mycoplasma contamination by polymerase chain reaction. Both parasitized and unparasitized erythrocytes were counted by flow cytometry, and compared regarding their size and internal complexity to the counting beads and PBMCs. Aliquots were made and stored at -8°C until needed for PBMCs stimulation.

### PBMCs stimulation

Malaria antigens differ in their capabilities to stimulate PBMCs: intact parasitized red blood cells (pRBC) are capable of inducing more rapid and intense pro-inflammatory responses from PBMCs than freeze-thaw lysates of *P. falciparum *[26]. To simulate *in vivo *malaria-specific responses as closely as possible, *P. falciparum *pRBC were used, with an adapted protocol for stimulation of PBMCs by Jeurink *et al *[22]. In brief, PBMCs were cultured at 10^6 ^cells/well in sterile polystyrene 48-well plates with flat-bottom wells (Corning Inc, Corning, NY, USA). Based on initial optimization experiments, aliquots of pRBC were thawed and cultured with PBMCs in IMDM with glutamax containing Yssel's supplements [27] with 2% human AB serum, 1% penicillin/streptomycin and 1% fungizone (Gibco-BRL), at a PBMCs:pRBC ratio of 1:2. PBMCs were also cultured under similar conditions with unparasitized erythrocytes (uRBC) (2 × 10^6 ^cells/well) as a negative control, and with soluble antibodies to CD3 and soluble antibodies to CD28 (Cat. No.555336 and 555725, Becton-Dickinson, Alphen aan den Rijn, The Netherlands) as a positive control. Monoclonal anti-CD3 and anti-CD28 antibodies provide co-stimulatory signals and polyclonal stimulation required for maximal proliferation of T lymphocytes [22,28]. Cell culture plates were incubated at 37°C in a humidified atmosphere containing 5% CO_2_. After PBMCs culturing for 1 day, we aspirated 75 μL of the supernatant per well to measure cytokine concentrations.

### Measurement of cytokine concentrations

Concentrations of IL-1β, IL-10, IL-12p70 and TNF were determined on a FACSCanto II flow cytometer by cytometric bead array system and analysed with FCAP software (all from Becton-Dickinson).

### Statistical analysis

Data were entered and analysed using SPSS for Windows (version 15.0. SPSS Inc., Chicago, IL, USA). Zinc deficiency and low zinc status were defined as plasma zinc concentrations < 9.9 μmol/L and < 10.7 μmol/L, respectively. Low magnesium status was defined by magnesium concentration < 750 μmol/L [29,30]. Iron deficiency anaemia was defined by co-existing iron deficiency (plasma ferritin concentration < 12 μg/L) and anaemia (haemoglobin concentration < 110 g/L). Fisher's Exact Test was performed to determine the association between inflammation (CRP levels) and sex, age, malaria status as well as nutritional status. Cytokine concentrations were ln-transformed to obtain normally-distributed values. Group differences in these values were analysed assuming t-distributions. Interactions between malaria and micronutrient indicators were assessed using multiple linear regression models on log-transformed cytokine data; the resulting effect sizes were exponentiated and expressed as percentage values. Linear regression analyses were also carried out to explore the associations between IL-1β, TNF and IL-10, and to what extent these associations were influenced by nutrient status and malaria infection status. The association between concentrations of TNF and IL-10 were considered as a measure of balance between the pro-inflammatory responses and the regulatory response. The analyses of the cytokine responses to pRBC are reported. As expected, the average response to uRBC (negative control) was less than to pRBC, whereas the average response to CD3/CD28 (positive control) was higher. Correction for these responses does not change the estimates of the associations between nutrient status and cytokine responses, or between malarial infection status and cytokine responses.

## Results

### General characteristics of the study population

**Figure 1:**
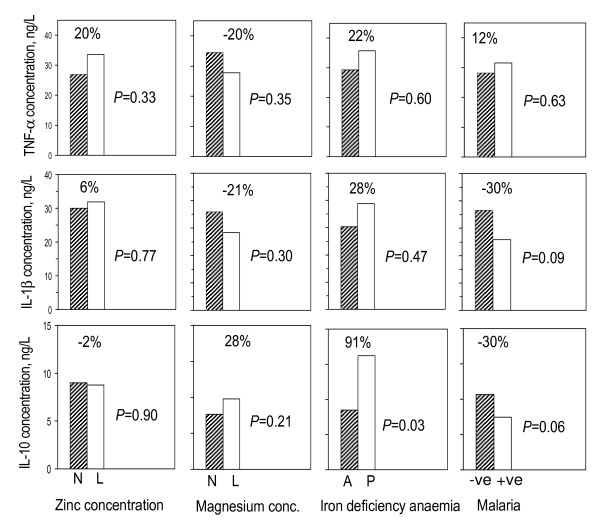
**Associations between nutrient status and supernatant cytokine concentrations, and between malaria infection status of the child at the time of blood collection and supernatant cytokine concentrations, following 24 h of PBMC stimulation with *Plasmodium falciparum*-infected erythrocytes**. N: normal concentrations; L: low concentrations (plasma concentrations of zinc and magnesium < 9.9 μmol/L and < 750 μmol/L, respectively); A: absence of iron deficiency anaemia; P: presence of iron deficiency anaemia (co-existing iron deficiency; plasma ferritin concentration < 12 μg/L and anaemia; haemoglobin concentration < 110 g/L). -ve: malaria negative; +ve: malaria positive. Percentages indicate paired group differences in supernatant cytokine concentrations, expressed as percentages relative to values observed in groups with normal plasma zinc or magnesium concentrations. *P*-values were obtained by assessing by multivariate analysis to what extent the proportional change in cytokine concentration that is associated with nutrient or malaria status.

Peripheral blood was collected from 135 boys and 169 girls; these had similar age distributions. The following prevalence values (n) were found: low zinc status: 63.1% (188); zinc deficiency: 48.3% (144); low magnesium status: 65.1% (194); iron deficiency anaemia: 9.4% (26); malaria: 46.1% (140). Malaria and age were associated with inflammation (determines as CRP levels); however, there was no evidence that inflammation was associated with zinc deficiency, magnesium deficiency or iron deficiency anaemia (Fisher's Exact Test). Detailed characteristics of the study population by malarial infection status are summarised in Table 1. In addition, the associations between nutrient status and supernatant cytokine concentrations, and between malaria infection status of the child at the time of blood collection and supernatant cytokine concentrations, following 24 h of PBMCs stimulation with *P. falciparum*-infected erythrocytes are also summarized (Figure 1). Adjustment for age class, sex and/or magnesium deficiency did not lead to marked changes in the associations between zinc deficiency and supernatant cytokine concentrations shown in Figure 1; conversely, adjustment for age class, sex and/or zinc deficiency did not lead to marked changes in the associations between magnesium deficiency and those supernatant cytokine concentrations.

**Table 1:** Characteristics of the study population, by malarial infection status

	*Plasmodium*-infected	*Plasmodium*-uninfected	P-value
Sex			0.56
Male	65	70	
Female	75	94	
Age class			0.03
6-12 months	7	19	
12-24 months	18	31	
24-48 months	61	58	
48-72 months	54	55	
Zinc deficient^1^			0.49
Yes	63	81	
No	74	80	
Magnesium deficient^2^			0.63
Yes	87	107	
No	50	54	
Iron deficiency anaemia^3^			< 0.001
Yes	2	24	
No	138	140	

^1 ^Plasma zinc concentration < 9.9 μmol/L; ^2^plasma magnesium concentration < 750 μmol/L; ^3 ^anaemia (haemoglobin concentration < 110 g/L) and iron deficient (plasma ferritin concentration < 12 μg/L.

### Association between nutrient indicators and in vitro innate cytokine production, by malaria infection status at the time of blood collection

**Figure 2:**
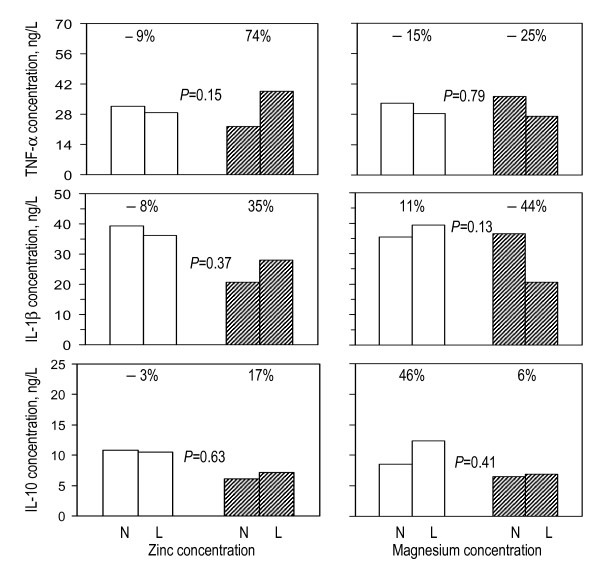
**Associations between nutrient status and supernatant cytokine concentrations following 24 h of PBMC stimulation with *Plasmodium falciparum*-infected erythrocytes, by malaria infection status of the child at the time of blood collection**. N: Normal concentrations; L: low concentrations (plasma concentrations of zinc and magnesium < 9.9 μmol/L and < 750 μmol/L, respectively). Data from children without and with malaria infection at the time of blood collection are indicated with open and shaded columns, respectively. Percentages indicate group differences in supernatant cytokine concentrations, expressed as percentages relative to values observed in groups with normal plasma zinc or magnesium concentrations. *P*-values were obtained by assessing by multivariate analysis to what extent the proportional change in cytokine concentration that is associated with nutrient status is different between children with and without malarial infection. The number of individuals with iron deficiency and malaria (table 1) was too small to meaningfully compare among groups.

When analysing IL-10 concentrations, all individuals with IL-10 concentrations below the detection limit were excluded. In some instances, differences in cytokine concentrations between nutrient replete and deficient children (Figure 2) seemed to depend on malarial infection status at the time of blood collection (Table 2). The profile of supernatant cytokine concentration appeared different between subjects with deficiencies in zinc, magnesium and with iron deficiency anaemia. In the absence of malaria infection at the time of blood collection, zinc deficiency was associated with marginal reductions in concentrations of TNF, IL-1β and IL-10. Amongst donors with malaria infection at the time of blood collection, zinc status was not associated with altered concentrations of IL-1β or IL-10, but low plasma zinc concentrations were associated with an increase in TNF concentration by 74% (11% to 240%, 95% CI). Malaria infection at the time of blood collection seemed to determine the magnitude of the association between low plasma zinc concentration and TNF concentration (9% reduction in children without malaria, as compared to 74% increase in their peers with malaria; although the statistical evidence for this difference was weak (*P *= 0.15).

**Table 2:** Influence of nutritional indicators on innate cytokines responses after 24-h *in vitro *stimulation of PBMCs with malaria-infected erythrocytes

Nutrient status indicator	Supernatant concentration (ng/L) after 24 h of stimulation					
	
	TNF-α		IL-1β		IL-10	
**Children without *Plasmodium *infection**						
Plasma zinc concentration						
Normal	31.7	(32)	39.3	(37)	10.8	(45)
Low	28.8	(28)	36.2	(36)	10.5	(20)
Difference	-9% (-51% to 67%)		-81% (-49% to 68%)		-3% (-42% to 63%)	
Plasma magnesium concentration						
Normal	33.2	(25)	35.5	(30)	8.5	(18)
Low	28.4	(35)	39.4	(43)	12.4	(27)
Difference	-15% (-54% to 59%)		11% (-40% to 104%)		46% (13% to 144%)	
Iron deficiency anaemia						
Absent	27.1	(51)	35.3	(63)	9.3	(37)
Present	33.9	(11)	43.8	(13)	19.9	(8)
Difference	25% (-45% to 183%)		24% (-42% to 170%)		113% (13% to 301%)	

**Children with *Plasmodium *infection**						
Plasma zinc concentration						
Normal	22.2	(27)	20.7	(27)	6.1	(12)
Low	38.7	(31)	28.0	(35)	7.2	(18)
Difference	74% (11% to 240%)		35% (26% to 146%)		17% (-34% to 107%)	
Plasma magnesium concentration						
Normal	36.3	(19)	36.5	(19)	6.5	(12)
Low	27.2	(39)	20.6	(43)	6.9	(18)
Difference	-25% (-64% to 55%)		-44% (-70% to 6%)		6% (-40% to 87%)	
Iron deficiency anaemia						
Absent	31.6	(61)	26.1	(64)	7.6	(32)
Present	64.9	(1)	17.7	(2)	3.2	(1)

Difference	Not calculated		-32% (-88% to 270%)		Not calculated	

Values indicate geometric means (n) or effect [95% CI]. Cut-off values to define low plasma concentrations of nutritional indicators: see text. When computing differences between values, the category with normal plasma concentration was used as the reference. 'With malaria' and 'without malaria' refers to infection status of children studied at the time of blood collection.

Magnesium deficiency, on the other hand, was associated with increased concentrations of IL-10; this increase was 46% in children without malaria, as compared to only 6% in their peers with malaria (Figure 2). Low magnesium concentrations seemed associated with reduced concentrations of TNF and IL-1β by -25% (95% CI: -64% to 55%; *P *= 0.79) and -44% (-70% to 6%; *P *= 0.13), respectively, in children with malaria infection at the time of blood collection, although these differences may have been due to chance. These results are a reverse of the situation in zinc deficiency. The numbers of individuals with both iron deficiency anaemia and malaria (Table 1) were too low to compare groups meaningfully.

### Influence of malaria and nutrient indicators on associations between cytokine concentrations

**Figure 3:**
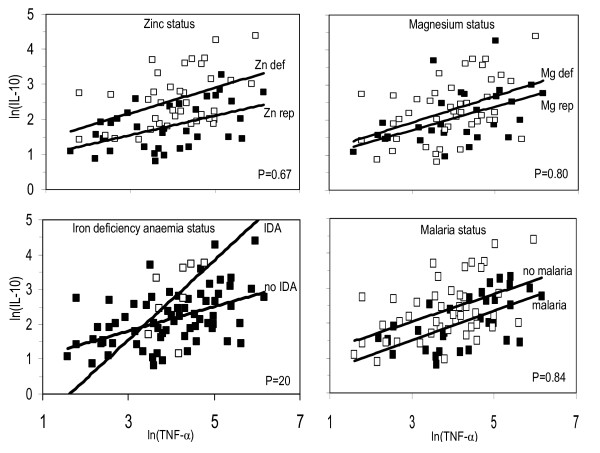
**Associations between supernatant concentrations of TNF- and IL-10 following 24 h stimulation of peripheral blood mononuclear cells with *Plasmodium falciparum*-infected erythrocytes, by micronutrient and malaria status at the time of blood collection**. Black blocks = zinc or magnesium replete, no iron deficiency anaemia or no malaria; open blocks = zinc and magnesium deficiency, iron deficiency anaemia or positive results for malaria tests at time of blood collection. *P*-values indicate probabilities of obtaining differences in associations between cytokine concentrations (as indicated by the slopes of the lines) as least as extreme as observed, assuming no differences.

No evidence was found that the associations between concentrations of TNF and IL-10 depended on zinc, magnesium or malaria status at time of blood collection, as indicated by differences in slopes of 17% (-44% to 147%; 95% CI, *P *= 0.67) for zinc status, 10% (-47% to 127%; 95% CI, *P *= 0.80) for magnesium status, or 3% (-24% to 39%; 95% CI, *P *= 84) for malaria (Figure 3). There was a tendency, albeit not significant, that iron deficiency anaemia (IDA) influenced the relationship between concentrations of TNF and IL-10, as indicated by the difference between slopes of 119% (35% to 637%; 95% CI, *P *= 0.20).

**Figure 4:**
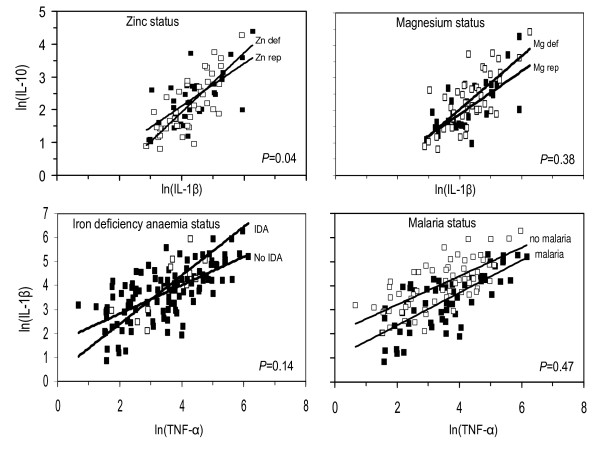
**Relationships between supernatant concentrations of TNF-α, IL-1β and IL-10 following 24 h stimulation of PBMCs with *Plasmodium falciparum*-infected erythrocytes, under different conditions of micronutrient and malaria status at the time of blood collection**. Black blocks = zinc replete, magnesium replete, or no malaria; open blocks = zinc deficiency or positive results for malaria test at time of blood collection.

Additional linear regression analyses (Figure 4) showed evidence that zinc status influenced the association between concentrations of IL-1β and IL-10, as indicated by differences in slopes of 118% (4% to 359%; 95% CI, *P *= 0.04). There was no evidence that malaria infection influenced the association between concentrations of TNF and IL-1β (Figure 4, malaria panel), as indicated by a difference in the slopes of regression lines of 9% (-13% to 35%; *P *= 0.47). In summary, these results show no evidence of influence on associations among innate cytokines, by the various conditions of micronutrient and malaria status at time of blood collection except for zinc status, for which there was some evidence that it influenced the association between IL-1β and IL-10. There was no evidence of an influence of micronutrient status and malaria on associations in other relationships. There were insufficient cases in all groups to explore and meaningfully compare the associations between IL-12 and other cytokines.

## Discussion

### Effects of plasma concentrations of mineral elements on in vitro cytokine responses by PBMCs

The biochemical data showed that most children in this study had nutrient deficiencies, particularly in zinc and magnesium and to a lesser extent iron deficiency anaemia. Zinc deficiency was associated with increased TNF responses in children with malaria infection at the time of blood collection but not in those without infection. TNF is a pro-inflammatory cytokine resulting in pathology if not properly regulated. In children with malarial infection, zinc deficiency was associated with increased production of IL-1β and IL-10, even if this increase did not bring the levels to those reached by individuals in the non-infected group. This is important because IL-10 is required to limit the production of pro-inflammatory cytokines, so that they do not lead to pathological consequences [31]. The low production of IL-10, however, could be due to the fact that the cytokine is said to be produced late (*in vivo*) following infection relatively to the innate cytokines. The initial production of TNF could also be the triggering factor by feedback mechanisms for production of IL-10 although Ramharter *et al *[32] reported increased responsiveness of *in vivo *primed cells as compared to malaria-naïve cells, with a tendency towards increased production of TNF. This can possibly explain the difference between subjects who were exposed or non-exposed at the time of blood collection, in response to *in vitro *stimulation in our study. These results show possible alterations in innate cytokine production particularly TNF and IL1-β due to the reported impaired macrophage functions and NK-cells activity in zinc deficiency [1,2,13,33]. Interaction between these cells leads to the production of innate cytokines in the early stages of infections.

The relatively higher cytokines levels in individuals with malarial infection as compared to their uninfected peers (Figure 2), however, can be explained by the priming of the immune system by malaria. Exposure of T cells to a plethora of Plasmodium antigens leads to priming, so that these cells during subsequent exposures even with subsets of these antigens can produce greatly increased amounts of IFN-γ. This cytokine is necessary for up-regulation of production of TNF and other pro-inflammatory cytokines by monocytes, but also Th subsets and even natural killer cells, in malaria infection [26,34]. The cellular source of these abundantly produced cytokines, like IL-1β and IL-10 remain to be established in future studies, as besides monocytes/macrophages and B-cells, also various T-cell subsets will be involved. The increase in innate cytokine production in zinc-deficient individuals with malarial infection can be the result of a shift in activated monocytes towards a pro-inflammatory immune response, associated increased levels of TNF and IL-1β, due to zinc deficiency in combination with prior priming of these cells due to previous exposure to malaria, as has been suggested before [35]. The initial contact with the pathogen directs towards production of pro-inflammatory cytokines to limit infection. Loharungsikul *et al *[36] proposed Toll-like receptors (TLRs) to play a role in innate immune recognition in which the differential expression of TLRs on antigen presenting cells (APCs) could be regulated by the *P. falciparum *parasite. This could account for the increase in levels of TNF in malaria-positive individuals regardless of micronutrient status (Figure 1). Glycosylphosphatidyl inositols (GPIs) that anchor *P. falciparum *merozoite surface protein 1 (MSP1) and merozoite surface protein 2 (MSP2) were described to be the pathogen associated molecular patterns (PAMPs) preferentially recognised by TLR-2 and TLR-4 [37]. The recognition and the interaction between these molecular patterns signal the induction of pro-inflammatory cytokine production. In addition, it is possible that parasite DNA attached to malarial pigment (haemozoin) produced in the course of infection further activates the innate immune response through TLR-9 engagement [38]. The expression of TLRs has been found to differ between malaria-infected and uninfected individuals, with higher expression being observed in infected patients [24,39]. These recent studies have further indicated TLR-2 to be highly expressed in mononuclear cells, particularly monocytes of *P. falciparum*-infected children and that TLR-2 are well responsive following stimulation with pRBCs resulting into stronger signals with consequential change in cytokine production profiles.

Unfortunately, the design of this study did not allow to ascertain the duration of infection at the time of blood collection. The children investigated may have been infected for some time, and may not have relied on the innate cytokines measured to control infection at the time of blood collection. It is also important to note that this concerned a cross-sectional study, that samples that were collected in a single time point, and that cytokines measured had accumulated in supernatant following 24-h stimulation of cultured cells. The design of our study does not allow time-dependent production of cytokines, or to establish specific cell subsets are sources of the cytokines measured.

The interesting result in this study is that the impact of magnesium deficiency on early cytokine responses followed a different profile from that observed with zinc status. Magnesium deficiency seemed to be associated overall with low TNF concentrations, low concentrations of IL-1β and higher concentrations of IL-10 in uninfected but not infected donors (Figure 2). Low levels of pro-inflammatory cytokines in malaria are critical because they reduce the ability of the initial innate immune response to limit infection. These results imply that magnesium deficiency directs early cytokine responses towards anti-inflammatory rather than pro-inflammatory cytokine responses, although further studies are still needed to confirm this hypothesis. The significantly increased IL-10 and variable alteration in levels of TNF and IL-1β in both malaria-negative and malaria-positive subjects with magnesium deficiency may explain the imbalance in cytokine production as a result of magnesium deficiency modulated by malaria status.

Methodological differences may explain contradictions between our findings and those from previous studies [7,9,40]. Parasitized erythrocytes were used to simulate the *in vivo *infection, whereas others used mitogens, lipopolysaccharides (LPS), phytohemagglutinin (PHA) and polyclonal stimulation. In addition, we used Ficoll-isolated PBMCs that had been stored for several months under frozen conditions, whereas whole blood stimulated within 15 minutes of collection was also used in some of the previous studies. McCall *et al *[24] stimulated freshly prepared PBMCs from adult naïve volunteers *ex vivo *with *P. falciparum *antigens. These findings suggest that zinc and other micronutrients can protect against malaria infection by a different means such as targeting specific pathogenic processes of infection *in vivo *[41]. Nevertheless, the idea that zinc can also reduce production of pro-inflammatory cytokines by inhibiting signal transduction in monocytes in healthy human subjects [42], particularly IL-1β and TNF [2,43,44], should be further explored. The latter idea is also supported by *in vitro *studies [45,46] in other conditions than malaria.

The results from this study and those conducted by others [47,48], IL-12 concentrations were below the detection limits. The most probable reason is the time required for maximal priming of pathogen recognition receptors (e.g. TLRs) on PBMCs by *P. falciparum*-parasitized erythrocytes. McCall *et al *[24] have shown that pro-inflammatory priming effects of *P. falciparum *require up to 48 hours to develop maximally, whereas we measured cytokines after 24 hours of stimulation. This priming is lacking in our culture system despite the reported poor *in vitro *induction of IL-12 by *P. falciparum *[49]. IL-12 levels obtained *in vitro *from stimulated monocytes and macrophages are generally low and zinc deficiency is reported to be associated with further decreased IL-12 production [50]. Early IL-12 activity is also liable to suppression by transforming growth factor (TGF)-β [51,52] that has been reported to variably influence and result in weak IL-12 activation and production, at least *in vivo*. Most of our donors responded towards production of IL-1β rather than TNF and IL-10. This is interesting since although different arguments reveal the pathological effect of IL-1β on cerebral malaria and severity of the disease in children [53], IL-1β together with other pro-inflammatory cytokines like IFN-γ and IL-6 is said to be protective against malaria by inducing parasite killing by monocytes, macrophages and neutrophils [54]. Production of IL-1β is induced by direct interaction between zinc and monocytes through activation of interleukine-1 receptor associated kinase (IRAK) which is dose-dependent [44]. Lower *in vivo *zinc levels, partially inhibit IRAK leading to diminished but not completely inhibited normal T-cells IL-β response. Results from this study may also reflect that stimulation of cryopreserved PBMCs by pRBCs results in a gradual production of innate cytokines preceded by IL-1β from the monocytes.

### Association between innate cytokines under different conditions of micronutrients and malaria status

The association between IL-1β and IL-10 was found to be influenced by zinc status (Figure 4). The two innate cytokines TNF and IL-1β are a prerequisite in early responses to malaria infection and IL-10 is an important regulatory cytokine affected by nutrient deficiencies and malaria infection status. This is critical under tropical situations where both micronutrients deficiencies and malaria prevail, posing a challenge to the early immune response to infections.

## Conclusions

In conclusion, it was shown micronutrient deficiencies to variably influence some *in vitro *innate cytokine concentrations. Zinc deficiency in particular, was found to possibly influence the *in vitro *production of various innate cytokines that particularly are modulated by malaria status. Magnesium deficiency, on the other hand, seemed to associate with higher concentrations of IL-10 in donors uninfected at time of blood collection. These results may be speculative indicators that while zinc deficiency and possibly iron deficiency anaemia might increase pro-inflammatory cytokines such as IL-1β and TNF, magnesium deficiency may have greater influence on anti-inflammatory cytokines such as IL-10. With regards to early innate cytokine responses to malaria, an ideal situation should be to supplement children with a combination of a few precisely selected micronutrients rather than single nutrients, although further studies involving larger sample sizes still need to be performed. This study has indicated the effect of poor nutrition on innate immune responses in children from malaria endemic area and how malaria infection may modulate these relationships. The findings have also shown plasticity in cytokine profiles of mononuclear cells reacting to malaria infection under conditions of different micronutrient deficiencies. Our findings therefore lay the foundations for future inclusion of selected micronutrients in malaria vaccine intervention programmes, particularly in developing countries, to boost immune response to malaria.

## Conflict of interests

The authors declare that they have no competing interests.

## Authors' contributions

EM carried out the protocol development and the laboratory analysis and drafted the manuscript. MM participated in cell culture protocol development, and laboratory analysis. JV: conducted the inclusion of the patients and the field work. MMcC: assisted in interpretation of data. JS and RO co-directed the field work; HV: conceived the study and acquired the funds, performed the statistical analysis and assisted in manuscript preparation. HS directed the study, supervised protocol development and manuscript preparation. All authors read and approved the final manuscript.

## References

[B1] Rink L Haase H Zinc homeostasis and immunity Trends Immunol 2007 28 1 4 17126599 10.1016/j.it.2006.11.005

[B2] Wellinghausen N Kirchner H Rink L The immunobiology of zinc Immunol Today 1997 11 519 521 10.1016/s0167-5699(97)01146-8 9386346

[B3] Wieringa FT Dijkhuizen MA West CE Ven-Jongekrijg J van der Muhilal Meer JWM van der Reduced production of immunoregulatory cytokines in vitamin A- and zinc-deficient Indonesian infants Eur J Clin Nutr 2004 58 1498 1504 15162133 10.1038/sj.ejcn.1601998

[B4] Black RE Therapeutic and preventive effects of zinc on serious childhood infectious diseases in developing countries Am J Clin Nutr 1998 68 476S 479S 9701163 10.1093/ajcn/68.2.476S

[B5] Muller O Becher H van Zweeden AB Ye Y Diallo DA Konate AT Gbangou A Kouyate B Garenne M Effect of zinc supplementation on malaria and other causes of morbidity in west African children: randomised double blind placebo controlled trial BMJ 2001 322 1567 11431296 10.1136/bmj.322.7302.1567 PMC33513

[B6] Shankar AH Genton B Baisor M Paino J Tamja S Adiguma T Wu L Rare L Bannon D Tielsch JM Alpers MP West KP Jr The influence of zinc supplementation on morbidity due to *Plasmodium falciparum*: a randomized trial in preschool children in Papua New Guinea Am J Trop Med Hyg 2000 62 663 669 11304051 10.4269/ajtmh.2000.62.663

[B7] Shankar AH Prasad AS Zinc and immune function: the biological basis of altered resistance to infection Am J Clin Nutr 1998 68 447S 463S 9701160 10.1093/ajcn/68.2.447S

[B8] Percival SS Neutropenia caused by copper deficiency: possible mechanisms of action Nutr Rev 1995 53 59 66 7770185 10.1111/j.1753-4887.1995.tb01503.x

[B9] Erickson KL Medina EA Hubbard NE Micronutrients and Innate Immunity J Infect Dis 2000 182 S5 S10 10944478 10.1086/315922

[B10] Walther M Woodruff J Edele F Jeffries D Tongren JE King E Andrews L Bejon P Gilbert SC De Souza JB Sinden R Hill AV Riley EM Innate immune responses to human malaria: heterogeneous cytokine responses to blood-stage *Plasmodium falciparum *correlate with parasitological and clinical outcomes J Immunol 2006 177 5736 5745 17015763 10.4049/jimmunol.177.8.5736

[B11] Keen CL Gershwin ME Zinc deficiency and immune function Annu Rev Nutr 1990 10 415 431 2200472 10.1146/annurev.nu.10.070190.002215

[B12] Walker CF Black RE Zinc and the risk for infectious disease Ann Rev Nutr 2004 24 255 275 10.1146/annurev.nutr.23.011702.073054 15189121

[B13] Ibs K-H Rink L Zinc-Altered Immune Function J Nutr 2003 133 1452S 1456S 12730441 10.1093/jn/133.5.1452S

[B14] Rink L Gabriel P Extracellular and immunological actions of zinc BioMetals 2001 14 367 383 11831466 10.1023/a:1012986225203

[B15] McKay I Rosen FS Innate immunity N Engl J Med 2000 343 338 344 10922424 10.1056/NEJM200008033430506

[B16] Stevenson MM Riley EM Innate immunity to malaria Nat Rev Immunol 2004 4 169 180 15039754 10.1038/nri1311

[B17] Pallauf J Rimbach G Nutritional significance of phytic acid and phytase Arch Animal Nutr 1997 50 301 319 10.1080/17450399709386141 9345595

[B18] Veenemans J Andang'o PEA Mbugi EV Kraaijenhagen RJ Mwaniki DL Mockenhaupt FP Roewer S Olomi RM Shao JF Meer JWM van der Savelkoul HFJ Verhoef H α^+^-Thalassemia protects against anemia associated with asymptomatic malaria: evidence from community-based surveys in Tanzania and Kenya J Infect Dis 2008 198 401 408 18582194 10.1086/589884

[B19] Moody A Rapid diagnostic tests for malaria parasites Clin Microbiol Rev 2002 15 66 78 11781267 10.1128/CMR.15.1.66-78.2002 PMC118060

[B20] Piper R Lebras J Wentworth L Hunt-Cooke A Houzé S Chiodini P Makler M Immunocapture diagnostic assays for malaria using *Plasmodium *lactate dehydrogenase (pLDH) Am J Trop Med Hyg 1999 60 109 18 9988333 10.4269/ajtmh.1999.60.109

[B21] Ryan A Analysis of blood serum on the Liberty Series II ICP-AES with the axially-viewed plasma ICP-24: Varian Inc 1998 1 9

[B22] Jeurink PV Vissers YM Rappard B Savelkoul HFJ T cell responses in fresh and cryopreserved peripheral blood mononuclear cells: Kinetics of cell viability, cellular subsets, proliferation, and cytokine production Cryobiol 2008 57 91 103 10.1016/j.cryobiol.2008.06.002 18593572

[B23] Hartgers FC Obeng BB Kruize YCM Dijkhuis A McCall M Sauerwein RW Luty AJF Boakye DA Yazdanbakhsh M Responses to malarial antigens are altered in helminth-infected children J Inf Dis 2009 199 1528 1535 10.1086/598687 19392626

[B24] McCall MBB Netea MG Hermsen CC Jansen T Jacobs L Golenbock D Ven AJAM van der Sauerwein RW *Plasmodium falciparum *infection causes proinflammatory priming of human TLR responses J Immunol 2007 179 162 171 17579034 10.4049/jimmunol.179.1.162

[B25] Rivadeneira EM Wasserman M Espinal CT Separation and concentration of schizonts of *Plasmodium falciparum *by Percoll gradients J Eukary Microbiol 1983 30 367 370 10.1111/j.1550-7408.1983.tb02932.x 6313915

[B26] Artavanis-Tsakonas K Riley EM Innate immune response to malaria: rapid induction of IFN-γ from human NK cells by live *Plasmodium falciparum*-infected erythrocytes J Immunol 2002 169 2956 2963 12218109 10.4049/jimmunol.169.6.2956

[B27] Yssel H De Vries JE Koken M Van Blitterswijk W Spits H Serum-free medium for generation and propagation of functional human cytotoxic and helper T cell clones J Immunol Methods 1984 72 219 227 6086760 10.1016/0022-1759(84)90450-2

[B28] Imada M Simons FE Jay FT HayGlass KT Antigen mediated and polyclonal stimulation of human cytokine production elicit qualitatively different patterns of cytokine gene expression Int Immunol 1995 7 229 237 7537536 10.1093/intimm/7.2.229

[B29] Lichodziejewska MDB Kos MDJ Rezler MDJ Grudzka MDK Duzzniewska MDM Budaj MDA Ceremuzynski MDPL Clinical symptoms of mitral valve prolapse are related to hypomagnesemia and attenuated by magnesium supplementation Am J Cardiol 1997 79 768 772 9070556 10.1016/s0002-9149(96)00865-x

[B30] Naderi ASA Reilly RF Hereditary etiologies of hypomagnesemia Nat Clin Pract Neph 2008 4 80 89 10.1038/ncpneph0680 18227801

[B31] Perkins DJ Weinberg JB Kremsner PG Reduced interleukin-12 and Transforming Growth Factor-beta in severe childhood malaria: relationship of cytokine balance with disease severity J Infect Dis 2000 182 988 992 10950804 10.1086/315762

[B32] Ramharter M Willheim M Winkler H Wahl K Lagler H Graninger W Winkler S Cytokine profile of *Plasmodium falciparum*-specific T cells in non-immune malaria patients Parasite Immunol 2003 25 211 219 12940964 10.1046/j.1365-3024.2003.00628.x

[B33] Wintergerst ES Maggini S Hornig DH Contribution of selected vitamins and trace elements to immune function Ann Nutr Metab 2007 51 301 323 17726308 10.1159/000107673

[B34] Artavanis-Tsakonas K Tongren JE Riley EM The war between the malaria parasite and the immune system: immunity, immunoregulation and immunopathology Clin Exp Immunol 2003 133 145 152 12869017 10.1046/j.1365-2249.2003.02174.x PMC1808775

[B35] Prasad A Zinc: mechanisms of host defense J Nutrition 2007 137 1345 1349 10.1093/jn/137.5.1345 17449604

[B36] Loharungsikul S Troye-Blomberg M Amoudruz P Pichyangkul S Yongvanitchit K Looareesuwan S Mahakunkijcharoen Y Sarntivijai S Khusmith S Expression of Toll-like receptors on antigen-presenting cells in patients with falciparum malaria Acta Trop 2008 105 10 15 17854755 10.1016/j.actatropica.2007.08.002

[B37] Krishnegowda G Hajjar AM Zhu J Douglass EJ Uematsu S Akira S Woods AS Gowda DC Induction of proinflammatory responses in macrophages by the glycosylphosphatidylinositols of *Plasmodium falciparum*: cell signaling receptors, glycosylphosphatidylinositol (GPI) structural requirement, and regulation of GPI activity J Biol Chem 2005 280 8606 8616 15623512 10.1074/jbc.M413541200 PMC4984258

[B38] Parroche P Lauw FN Goutagny N Latz E Monks BG Visintin A Halmen KA Lamphier M Olivier M Bartholomeu DC Gazzinelli RT Golenbock DT Malaria hemozoin is immunologically inert but radically enhances innate responses by presenting malaria DNA to Toll-like receptor 9 Proc Nat Acad Sci USA 2007 104 1919 1924 17261807 10.1073/pnas.0608745104 PMC1794278

[B39] Hartgers FC Obeng BB Voskamp A Larbi IA Amoah AS Luty AJF Boakye D Yazdanbakhsh M Enhanced Toll-Like Receptor responsiveness associated with mitogen-activated protein kinase activation in *Plasmodium falciparum*-infected children Infect Immun 2008 76 5149 5157 18710867 10.1128/IAI.01579-07 PMC2573356

[B40] Rivera MT De Souza AP Araujo-Jorge TC De Castro SL Vanderpas J Trace elements, innate immune response and parasites Clin Chem Lab Med 2003 41 1020 1025 12964807 10.1515/CCLM.2003.156

[B41] Overbeck S Rink L Haase H Modulating the immune response by oral zinc supplementation: a single approach for multiple diseases Arch Immunol Ther Exp 2008 56 15 30 10.1007/s00005-008-0003-8 PMC7079749 18250973

[B42] Haase H Rink L Signal transduction in monocytes: the role of zinc ions BioMetals 2007 20 579 585 17453150 10.1007/s10534-006-9029-8

[B43] Prasad AS Bao B Beck FWJ Kucuk O Sarkar FH Antioxidant effect of zinc in humans Free Radic Biol Med 2004 37 1182 1190 15451058 10.1016/j.freeradbiomed.2004.07.007

[B44] Wellinghausen N Martin M Rink L Zinc inhibits interleukin-1-dependent T cell stimulation Eur J Immunol 1997 27 2529 2535 9368606 10.1002/eji.1830271010

[B45] von Bulow V Dubben S Engelhardt G Hebel S Plumakers B Heine H Rink L Haase H Zinc-dependent suppression of TNF-alpha production is mediated by protein kinase A-induced inhibition of Raf-1, I kappa B kinase beta, and NF-kappa B J Immunol 2007 179 4180 4186 17785857 10.4049/jimmunol.179.6.4180

[B46] von Bulow V Rink L Haase H Zinc-mediated inhibition of cyclic nucleotide phosphodiesterase activity and expression suppresses TNF-alpha and IL-1beta production in monocytes by elevation of guanosine 3',5'-cyclic monophosphate J Immunol 2005 175 4697 4705 16177117 10.4049/jimmunol.175.7.4697

[B47] Keller CC Yamo O Ouma C Ong'echa JM Ounah D Hittner JB Vulule JM Perkins DJ Acquisition of hemozoin by monocytes down-regulates interleukin-12 p40 (IL-12p40) transcripts and circulating IL-12p70 through an IL-10-dependent mechanism: *in vivo *and *in vitro *findings in severe malarial anemia Infect Immun 2006 74 5249 5260 16926419 10.1128/IAI.00843-06 PMC1594872

[B48] Uh HW Hartgers FC Yazdanbakhsh M Houwing-Duistermaat JJ Evaluation of regression methods when immunological measurements are constrained by detection limits BMC Immunol 2008 9 59 18928527 10.1186/1471-2172-9-59 PMC2592244

[B49] Scragg IG Hensmann M Bate CAW Kwiatkowski D Early cytokine induction by *Plasmodium falciparum*is not a classical endotoxin-like process Eur J Immunol 1999 29 2636 2644 10458778 10.1002/(SICI)1521-4141(199908)29:08<2636::AID-IMMU2636>3.0.CO;2-Y

[B50] Prasad AS Effects of zinc deficiency on Th1 and Th2 cytokine shifts J Inf Dis 2000 182 S62 68 10.1086/315916 10944485

[B51] Omer FM Kurtzhals JAL Riley EM Maintaining the immunological balance in parasitic infections: a role for TGF-β? Parasitol Today 2000 16 18 23 10637583 10.1016/s0169-4758(99)01562-8

[B52] Torre D Speranza F Martegani R Role of proinflammatory and anti-inflammatory cytokines in the immune response to *Plasmodium falciparum *malaria Lancet Infect Dis 2002 2 719 720 12467687 10.1016/s1473-3099(02)00449-8

[B53] Clark IA Rockett KA The cytokine theory of human cerebral malaria Parasitol Today 1994 10 410 412 15275552 10.1016/0169-4758(94)90237-2

[B54] Perlmann P Troye-Blomberg M Malaria and the immune system in humans Chem Immunol 2002 80 229 242 12058641 10.1159/000058846

